# Understanding dental pulp inflammation: from signaling to structure

**DOI:** 10.3389/fimmu.2024.1474466

**Published:** 2024-10-29

**Authors:** Sandra Pohl, Tobias Akamp, Martyna Smeda, Stefan Uderhardt, David Besold, Gabriel Krastl, Kerstin M. Galler, Wolfgang Buchalla, Matthias Widbiller

**Affiliations:** ^1^ Department of Conservative Dentistry and Periodontology, University Hospital Regensburg, Regensburg, Germany; ^2^ Medical Department 3, Rheumatology and Immunology, University Hospital Erlangen, Erlangen, Germany; ^3^ Department of Conservative Dentistry and Periodontology, University Hospital Würzburg, Würzburg, Germany; ^4^ Department of Operative Dentistry and Periodontology, University Hospital Erlangen, Erlangen, Germany

**Keywords:** dental pulp, immunity, pulpitis, inflammation, dentin, dental caries, cytokines, chemokines

## Abstract

The pulp is a unique tissue within each tooth that is susceptible to painful inflammation, known as pulpitis, triggered by microbial invasion from carious lesions or trauma that affect many individuals. The host response involves complex immunological processes for pathogen defense and dentin apposition at the site of infection. The interplay of signaling between the immune and non-immune cells via cytokines, chemokines, neuropeptides, proteases, and reactive nitrogen and oxygen species leads to tissue reactions and structural changes in the pulp that escalate beyond a certain threshold to irreversible tissue damage. If left untreated, the inflammation, which is initially localized, can progress to pulpal necrosis, requiring root canal treatment and adversely affecting the prognosis of the tooth. To preserve pulp vitality and dental health, a deeper understanding of the molecular and cellular mechanisms of pulpitis is imperative. In particular, elucidating the links between signaling pathways, clinical symptoms, and spatiotemporal spread is essential to develop novel therapeutic strategies and push the boundaries of vital pulp therapy.

## 1 Introduction

Inflammation is a cornerstone of both medical and dental practice. Historical descriptions date back thousands of years and were characterized by observable signs such as redness, swelling, pain and warmth in infected wounds, later defined along with dysfunction as the cardinal signs of inflammation (1). These observations continue to shape modern medical terminology, as evidenced by the ancient Greek and Latin terms “phlegmon” and “inflammation”, which describe the fiery and rapidly spreading nature of inflammatory reactions, such as those experienced by toothache (2). While research into inflammation initially focused on acute responses, the focus has shifted to chronic inflammation, particularly in relation to systemic diseases such as type 2 diabetes, atherosclerosis, asthma, cancer and neurodegenerative diseases (3).

In general, the inflammatory response is the body’s defense against harmful stimuli such as infection and tissue damage. Its aim is to eliminate the cause of the inflammation and restore tissue homeostasis (4). This intricate process involves the orchestrated recruitment of immune cells and plasma components to the affected site, blending rapid innate responses with adaptive immunity (5).

Thereby, the inflammatory reaction involves several steps. First, the damaging stimuli are detected by sentinel cells such as macrophages, mast cells or dendritic cells in the tissue. Immune cells and plasma proteins are then recruited from the bloodstream to the site of inflammation, where they neutralize the triggers through phagocytosis or the release of cytotoxic compounds. Successful defense leads to anti-inflammatory and regenerative mechanisms that orchestrate tissue repair through a complex signaling network (4).

While an effective inflammatory response protects against damage, dysregulation or persistent stimulation can lead to adverse outcomes, including chronic inflammatory disease and tissue destruction (6). Oral health is no exception, with both acute and chronic inflammation posing a threat, particularly in conditions such as dental caries. The latter is a global health burden (7) resulting from bacterial biofilms that cause enamel and dentin degradation, potentially leading to painful inflammation within the dental pulp, which forms the connective tissue inside the tooth (8). The prevalence of pulp inflammation (pulpitis) with severe pain is high (9) and the most common reason for emergency dental care (10).

For many years, complete pulp removal followed by root canal treatment was the preferred therapy when symptoms were severe enough (11). More recently, however, a growing number of reports have advocated minimally invasive approaches with the aim of preserving all or part of the pulp, which has a positive effect on the functionality and long-term prognosis of the tooth (12). Understanding the physiology of pulp inflammation is essential for the development of innovative treatment strategies. Therefore, the aim of this review is to elucidate the mechanisms of the immune response in general and in the dental pulp in particular, in order to identify therapeutic approaches and to highlight unresolved scientific questions. The focus here will lie on signaling pathways in the dental pulp, structural changes due to immune responses and the resolution of inflammation.

The illustrations of structural transitions in the pulp tissue presented hereafter are based in part on Harold R. Stanley’s seminal textbook from 1976, originally illustrated in black and white, which covers the histology of the human pulp in healthy, pathological and healing conditions (13). Fortunately, the authors were provided with the original histological slides used in the book, which were meticulously restored and reproduced in color for better clarity and comprehension.

## 2 Pathogenesis of pulp inflammation

### 2.1 Anatomical and physiological characteristics

**Figure 1:**
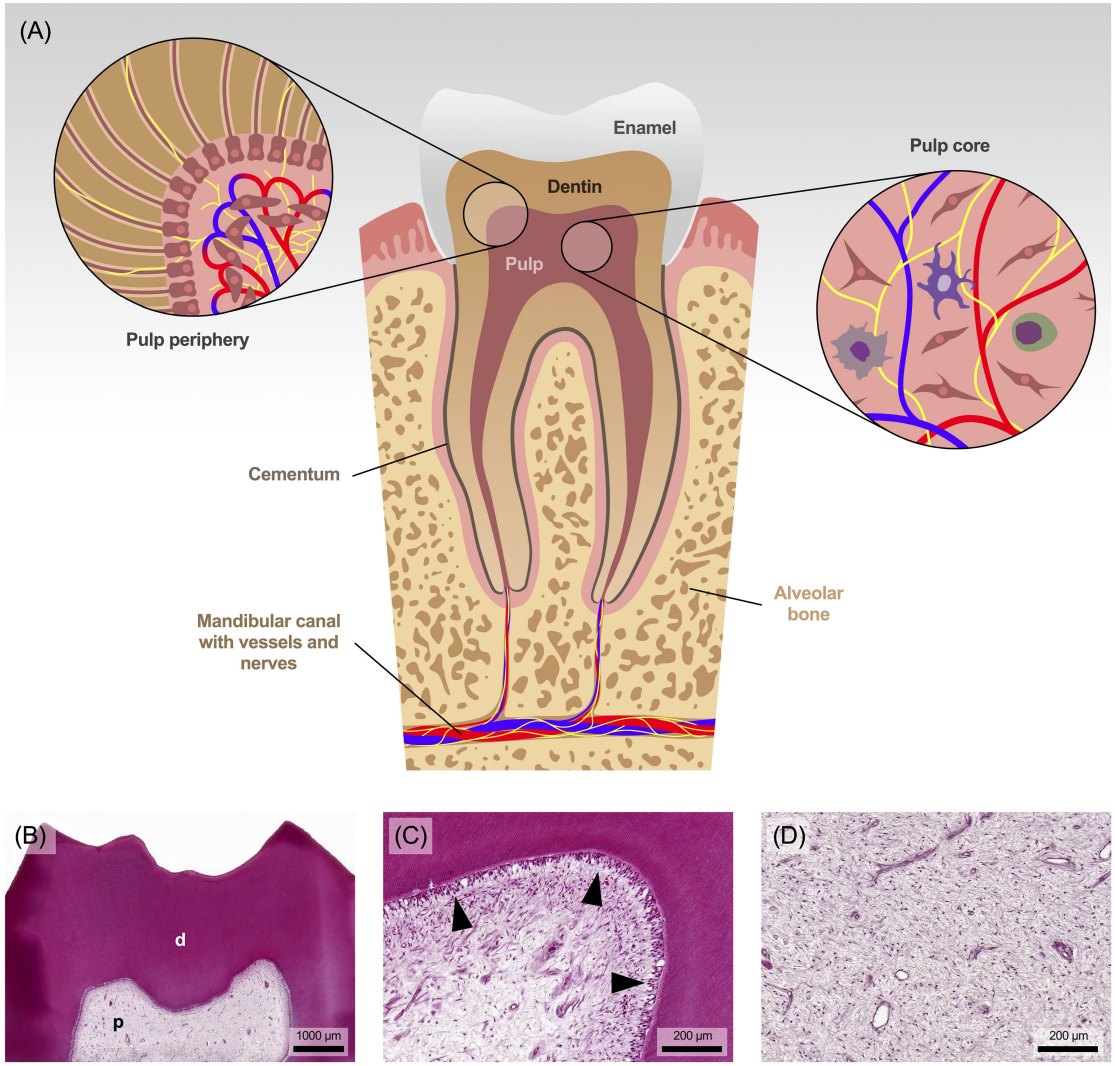
**(A)** Anatomical view of the tooth with pulp and surrounding area. **(B)** The pulp (p) is surrounded and protected by tubular dentin (d). **(C)** The dentin is lined by a single layer of dentin-forming cells, the odontoblasts (marked with arrowheads), followed in the pulp periphery by progenitor cells along with a capillary and nerve plexus. **(D)** The core of the pulp consists of collagenous connective tissue, rich in fibroblasts and containing some tissue-resident immune cells. Staining: hematoxylin and eosin.

Anatomically speaking, the pulp is undoubtedly unique in the human body, as it is completely enclosed by the hard structures of enamel, dentin and cementum (**Figures 1A, B**), and communicates with the environment only through the vessels and nerves that penetrate the small openings at the tip of the root (14). It is therefore mechanically well protected within the tooth but has no means of spatial expansion. Functionally and developmentally, the pulp and dentin form a closely linked unit called pulp-dentin-complex, which derives from the cranial neural crest (15).

Specialized cells known as odontoblasts form a continuous layer at the periphery of the pulp, adjacent to the dentin (**Figure 1C**). Due to their anatomical position, they are the first cells to encounter noxious stimuli and play a pivotal role in sensing those signals and mediating tissue responses (8, 16). During tooth development and use, these cells produce a collagenous scaffold called predentin, which gradually mineralizes into dentin. A thin cell process from each odontoblast remains embedded in the mineralized matrix, resulting in the tubular structure of dentin. However, the pulp core, rich in fibroblasts, collagen, hyaluronan, proteoglycan and water (**Figure 1D**), resembles mesenchyme and gelatinous connective tissue (8, 14).

Entering the tooth from the alveolar bone through the apical foramen, a network of blood vessels and nerve fibers pervades the entire pulp. The nerve fibers branch extensively throughout the pulp chamber, forming the ‘Raschkow plexus’ beneath the odontoblast layer (14). They are primarily of trigeminal origin and include both myelinated Aδ fibers (10-30%) and unmyelinated C fibers (70-90%). As sensory nerve fibers they play a crucial role in detecting stimuli such as changes in temperature, but also pressure or tissue injury (17). Aδ fibers are concentrated in the peripheral region of the pulp, particularly near dentin and in the subodontoblastic layer, and transmit sharp, well-localized and stimulus-dependent sensations, whereas unmyelinated C fibers are located in the central region of the pulp tissue and are responsible for transmitting diffuse, dull and stimulus-persistent sensations associated with pain (15). The differentiation of pain quality, duration and stimulus reference is currently the key criterion for clinically distinguishing between reversible and irreversible inflammation, and thus determining the need for endodontic treatment.

In terms of cellular composition, the pulp tissue is dominated by pulp fibroblasts. Stem cells are also found in perivascular niches and have both mesenchymal and neural properties due to their ectodermal origin. In addition, even under physiological conditions, immune cells such as granulocytes, T cells (cluster of differentiation [CD]8+ and CD4+), monocytes/macrophages, dendritic cells and, to a lesser extent, NK cells, B cells and regulatory T cells are present in the pulp. In healthy pulp tissue, leukocytes (CD45+) represent approximately 1% of the entire pulp tissue, while granulocytes are the largest subpopulation (50%) followed by T lymphocytes (32%) and monocytes/macrophages (9%). Smaller subpopulations include dendritic cells (4%), natural killer cells (3%) and B lymphocytes (2%) (18). Together with odontoblasts, fibroblasts and stem cells, these tissue-resident immune cells contribute to the immunological capacity of the dental pulp (14, 18). During acute inflammation, the innate immune response is initiated, characterized by a significant increase in neutrophils and macrophages. As the inflammation becomes chronic, there is a notable rise in B cells. Although T cells are typically central to adaptive immunity, their levels may not significantly change during chronic pulpitis, however, the specific role of T cells in chronic pulpitis is not fully understood (19, 20).

### 2.2 Noxious stimuli for the dental pulp

Various factors such as bacterial invasion from caries, iatrogenic damage during dental procedures, exposure to irritating chemicals or traumatic injury can damage the dental pulp. These stimuli can cause acute inflammation that, if left untreated, can progress to chronic inflammation and, in severe cases, tissue necrosis. Besides the physical barrier provided to the pulp by the mineralized dental hard tissue, a continuous outward flow of fluid in the dentin tubules also provides a defense mechanism against potentially harmful substances and bacteria from the oral cavity (8). Short-term stimuli, such as invasive crown preparation or minor trauma, induce a brief acute inflammation, which is usually followed by tissue regeneration or repair. However, prolonged stimuli such as dental caries, which typically involve loss of hard tissue and microbial infection, lead to chronic inflammation and may result in pulp necrosis (21).

In caries, which is still a highly prevalent disease worldwide, acidic metabolites released by bacteria such as streptococci, lactobacilli and actinomycetes and other acidogenic species demineralize enamel and dentin (8). Even before the bacteria pass enamel and dentin to the pulp cavity, their metabolic products invade the pulp tissue and cause local immune reactions. If the bacterial invasion is not interrupted by caries excavation, it will eventually lead to pulp necrosis, allowing infection of the pulp cavity and root canals, ultimately causing apical periodontitis (22).

### 2.3 Triggers and sensors of the inflammatory response

In general, inflammatory reactions in the pulp can be triggered by exogenous or endogenous factors (**Table 1**). A common trigger for activating sensors in the pulp is caries, primarily involving gram-negative and gram-positive bacteria (23). Additionally, fungi such as *Candida albicans* are reported to form cross-kingdom synergisms with cariogenic bacteria and also possess cariogenic properties themselves (24). Furthermore, the involvement of viruses or phages present in the oral environment cannot be ruled out as they may act directly or indirectly by altering the biofilm composition (25). In addition to infectious aspects, tissue damage results in the release of cytosolic and nuclear substances from the affected cells, which can also activate specific receptors. Although a number of receptors are involved in pulp inflammation, research has predominantly focused on Toll-like receptors (TLRs), leaving other receptors and their ligands underexplored and in need of further investigation.

**Table 1:** Triggers and sensors of the inflammatory response (32, 104–106).

Ligand	Receptor	Possible sources
Pathogen-associated molecular patterns (exogenous)		
Lipopolysaccharide	TLR4	Gram-negative bacteria
Lipoteichoic acid	TLR2	Gram-positive bacteria
Flagellin	TLR5	Bacteria
Triacyl lipoproteins	TLR1, TLR2	
CpG motifs	TLR9	Bacteria and viruses
Viral dsRNA	RIG-I, MDA5	Viruses
Viral ssRNA	TLR7, TLR8	
Zymosan	TLR2	Fungi
β-Glucan	Dectin-1, TLR2, TLR4	
Damage-associated molecular patterns (endogenous)		
Adenosine triphosphate	P2X7, P2Y2	Cytosol of affected cells
Uric acid	NLRP3, P2X7	
Heat shock proteins	TLR2, TLR4, CD91	
S100 proteins	TLR2, TLR4, RAGE	
DNA	TLR9, AIM2	Nucleus of affected cells
RNA	TLR3, TLR7, TLR8, RIG-I, MDA5	
HMGB1	TLR2, TLR4, RAGE	
Histones	TLR2, TLR4	
Biglycan	TLR2, TLR4, NLRP3	Extracellular matrix of affected cells

Exogenously, microbial pathogens induce the response through specific pathogen-associated molecular patterns (PAMPs) that are recognized by pattern recognition receptors (PRRs) (26). These receptors, expressed by cells like macrophages, dendritic cells and fibroblasts, include transmembrane proteins such as TLRs and C-type lectin receptors (CLRs), and cytosolic proteins such as nucleotide-binding oligomerization domain (NOD)-like receptors (NLRs) and retinoic acid-inducible gene I (RIG-I)-like receptors (RLRs) (27). Activation of these receptors regulates pro-inflammatory gene transcription and complex immune signaling cascades (28). Alternatively, exogenous non-microbial triggers, such as allergens (e.g. silica) and irritant particles (e.g. asbestos) are detected by the NOD-, leucine rich repeat (LRR)- and pyrin domain-containing protein 3 (NLRP3) resulting in the formation and activation of the NLRP3 inflammasome (26, 29).

Endogenous signaling molecules from damaged, necrotic or stressed tissue, known as damage-associated molecular patterns (DAMPs), contribute to inflammation (**Table 1**). These include intracellular components such as adenosine triphosphate (ATP) and uric acid, which are released into the extracellular space due to loss of cell membrane integrity. PRRs are also activated by DAMPs to trigger an inflammatory response, although the exact role of TLRs remains controversial in the literature. While TLRs are known for detecting microbial molecules, they have also been reported to recognize DAMPs, such as tissue breakdown products or cell fragments, however, other studies have not supported these findings (26, 29).

## 3 Cellular players in pulpitis

### 3.1 Components of the immune response

**Figure 2:**
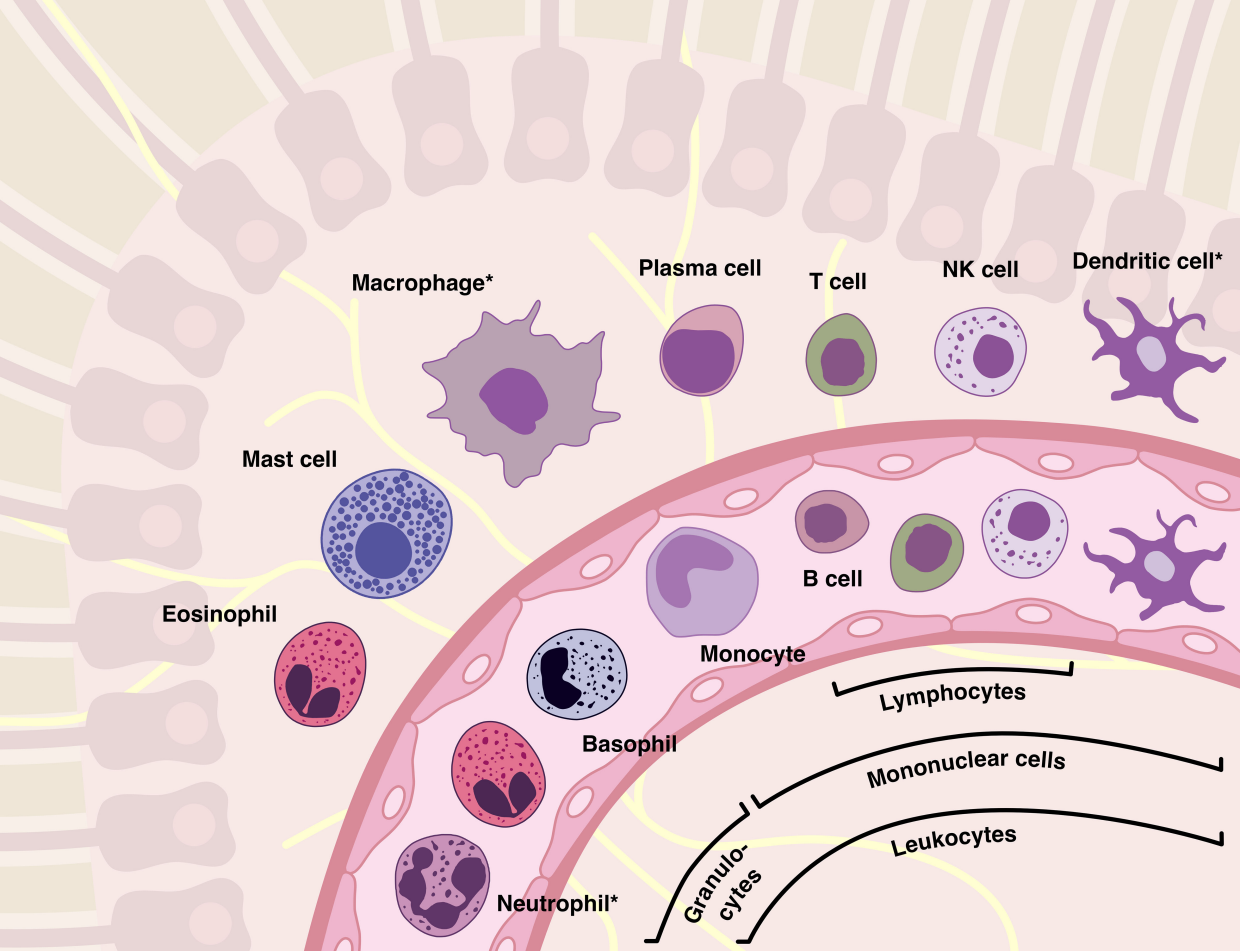
The majority of inflammatory cells originate from the blood and, as shown, have an equivalent in the tissues. Leukocytes can be categorized into several families based on their morphological and functional characteristics, such as granulocytes (“polymorphs”), mononuclear cells, lymphocytes, or phagocytes (marked with an asterisk). It should be noted that although basophilic granulocytes are similar to mast cells in many ways, they are not their precursor cells.

The inflammatory response involves several types of both tissue-specific and immune cells, with circulating leukocytes being of particular importance. These cells are transported throughout the body by the bloodstream, ensuring a rapid and widespread immunological response in all perfused tissues (28). Three populations of leukocytes can be distinguished: granulocytes, lymphocytes and monocytes (**Figure 2**). Lymphocytes and monocytes, characterized by their non-segmented nuclei, are collectively referred to as mononuclear cells. However, the term “granulocyte” is derived from the abundance of granules found in the cytoplasm.

Functionally, immune cells are involved in both the innate and adaptive immune response of the dental pulp. Innate immunity offers immediate defense against pathogens through general mechanisms like physical barriers and non-specific inflammatory responses, serving as the body’s first line of defense. Adaptive immunity, on the other hand, allows the body to recognize specific pathogens and develop a tailored response through antibody production and the activation of specific immune cells. These cells interact closely with the tissue-specific cells, odontoblasts and fibroblasts, to mount a targeted immune response.

#### 3.1.1 Neutrophil granulocytes

**Figure 3:**
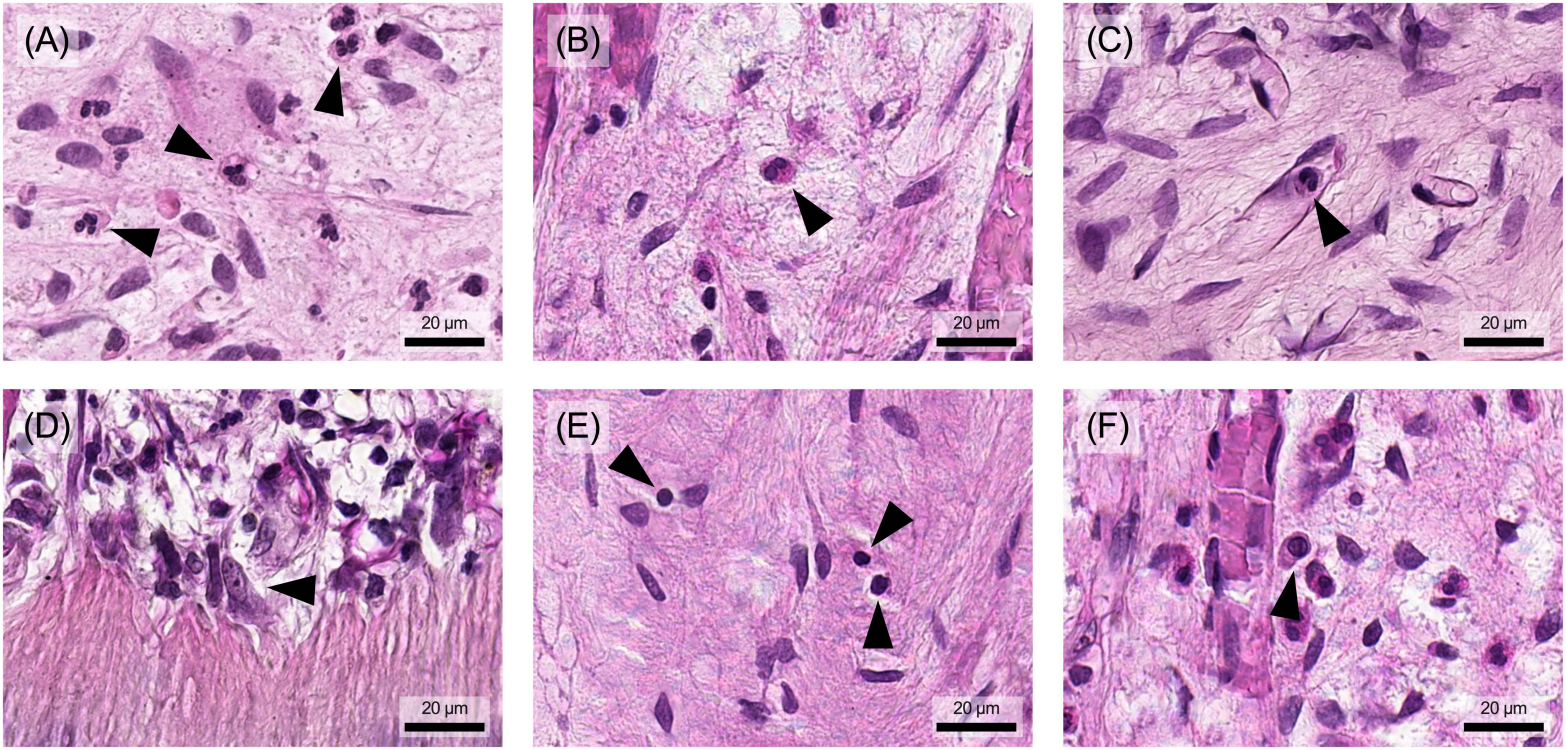
Different types of immune cells can be observed in human pulp tissue that has undergone inflammatory changes. **(A)** Neutrophil granulocytes and **(B)** eosinophil granulocytes are found in pulp tissue during inflammation. **(C)** Monocytes are found in blood vessels and differentiate into **(D)** macrophages as they invade the tissue. **(E)** Lymphocytes are also present and can be distinguished from pulp-typical fibroblasts by their shape, size, and nuclear appearance. **(F)** The plasma cells, derived from B lymphocytes, have a larger cell body with an asymmetrically positioned nucleus. Arrowheads indicate the different cell types described in each figure. Histological specimen from H. R. Stanley (13); staining: hematoxylin and eosin.

Neutrophil granulocytes are also known as polymorphonuclear granulocytes (PMNs) because they have differently segmented nuclei (**Figure 3A**). Another characteristic is the abundance of small granules in the cytoplasm, which contain a large number of enzymes that are crucial for their antimicrobial activity.

Neutrophil granulocytes play a key role in the acute inflammatory response as they are the initial cellular effectors of the body’s reaction to invading pathogens. Likewise, neutrophils are abundantly recruited to the pulp and are the first line of defense in the innate immune response to infection (18). Upon pathogen recognition, neutrophils must first migrate from the bloodstream to the target tissue site, which is regulated by both pro-inflammatory, e.g. tumor necrosis factor (TNF)-α and interleukin (IL)-1β, and chemotactic mediators, e.g. IL-8 and leukotriene B4 (30). Their antimicrobial activity is based on three mechanisms: first, they can take up and degrade pathogens as phagocytes (phagocytosis). Second, through degranulation, they release proteolytic enzymes and generate toxic reactive oxygen species (ROS) and reactive nitrogen species (RNS). This process can also damage host tissue as it is not specifically targeted at pathogens. Third, neutrophil granulocytes can produce neutrophil extracellular traps (NETs), intricate networks composed primarily of neutrophil DNA armed with antimicrobial peptides and enzymes. Pathogens become trapped in these structures and eventually fall victim to the increased concentration of antimicrobial agents.

However, the importance of neutrophil granulocytes in the inflammatory response goes beyond these antimicrobial effects. They can produce both pro- and anti-inflammatory cytokines, chemokines and other mediators, actively regulating the course of the inflammatory response (31). At 24 hours, the life cycle of neutrophils is short and predetermined by the initiation of apoptosis. Besides regulating population size, apoptosis also plays an important role in terminating the inflammatory response. Apoptotic neutrophilic granulocytes are eliminated by macrophages through non-phlogistic phagocytosis (efferocytosis), preventing the unintended release of toxic granule components. Following uptake of these apoptotic cells, macrophages cease production of proinflammatory mediators and begin to release anti-inflammatory and inflammation-resolving agents (32).

#### 3.1.2 Eosinophil granulocytes

Eosinophil granulocytes typically have a bilobed nucleus and relatively large, distinctly eosinophilic (red) granules (**Figure 3B**). They are multifunctional cells that accumulate primarily in tissues with mucosal surfaces and play an important role in defense against parasites, particularly worms. Eosinophil granulocytes were observed in both healthy and inflamed pulp tissue, however, they represent only a small proportion of immunocompetent cells (21, 33).

Their defense mechanism involves the release of toxic components (e.g. enzymes) from granules and the generation of ROS. When activated, they secrete cytokines, lipid mediators such as leukotrienes and platelet-activating factor (PAF), which have a pro-inflammatory effect. In addition, they can stimulate mast cells and basophilic granulocytes to degranulate, thereby contributing to the initiation and progression of an inflammatory response. They have also been implicated in the pathogenesis of allergic and neoplastic diseases (31).

#### 3.1.3 Basophil granulocytes

Basophil granulocytes have a relatively large and variably shaped nucleus, which is often covered by numerous large basophilic granules (34). After differentiating in the bone marrow, they circulate in the blood and are not usually found in connective tissues such as the dental pulp.

Through high-affinity immunoglobulin (Ig)E receptors on their surface, they release histamine stored in their granules upon cross-linking of bound IgE by antigens. They can also be activated by various cytokines, allergens and parasite products. A characteristic feature of basophil granulocytes is their ability to release cytokines and chemokines, to recruit other immune cells to the site of inflammation and to modulate the function of these cells (31). Additional functions of basophilic granulocytes include maintaining the memory function of humoral immunity, enhancing B cell proliferation and antibody production, and inducing helper T cell responses (35).

#### 3.1.4 Mast cells

Mast cells share cellular and functional similarities with basophilic granulocytes, but are tissue-resident cells. As mature cells, they are commonly found in the skin, mucous membranes and deeper connective tissues (32). So far, their occurrence has been described histologically in severe inflammatory conditions of the pulp (36). In general, mast cells play an important role in initiating and orchestrating both innate and adaptive immune responses against pathogens. They are crucial for immune surveillance and are strategically located at interfaces between the environment and the body, such as the skin or the gastrointestinal and respiratory tract. However, they are also found in close proximity to blood vessels and nerve fibers, allowing them to relay information about invading pathogens to these neighboring structures through the release of mediators (32).

Mast cells express a variety of PRRs on their surface, such as TLRs, to directly recognize pathogens and their PAMPs. In addition, they possess high affinity receptors for IgE, as well as receptors for IgG, which allow mast cells to be sensitized and activated. Upon activation, mast cells rapidly release pre-formed granular components, including histamine, proteases and TNF-α. They also synthesize pro-inflammatory mediators such as prostaglandins, leukotrienes, cytokines and chemokines. All this leads to the characteristic vascular changes associated with the inflammatory response and the chemotactic recruitment of leukocytes to the site of inflammation (31, 32).

#### 3.1.5 Monocytes and macrophages

Monocytes, the largest leukocytes in the bloodstream, typically have a kidney-shaped nucleus and blue-grey cytoplasm with blue granules (**Figure 3C**). Under the light microscope, macrophages appear relatively pale with a hint of eosinophilia, highlighting their large, bright and often irregularly shaped nucleus (**Figure 3D**) (31). These cells can be classified into tissue-specific subtypes, including inflammatory macrophages, microglia, Kupffer cells, osteoclasts and alveolar macrophages. Similarly, in pulp tissue, monocytes migrate from the bloodstream into the connective tissue, where they differentiate into large phagocytic macrophages or clastic cells (34). They are found as tissue-resident cells in healthy pulp tissue and, in particular, in inflamed pulp tissue (37, 38).

Monocytes and macrophages exhibit a high degree of functional versatility, playing critical roles in organogenesis, tissue maintenance and metabolic homeostasis (32). They also have a patrolling function and are essential in the initial host response (33). To recognize pathogens and PAMPs, monocytes and macrophages express a variety of PRRs on their surface. Following pathogen recognition, for example via TLRs, nuclear factor kappa B (NF-κB) facilitates the production of antimicrobial ROS and RNS as well as proinflammatory cytokines such as IL-1, TNF-α and IL-8 in monocytes and macrophages. This also leads to the recruitment and activation of other immune cells, such as T and B cells, promoting a more specific immune response. In addition, macrophages are antigen-presenting cells and can stimulate the functions of cytotoxic T cells and T helper cells.

However, the function of macrophages in the course of inflammatory processes is more diverse than expected. As described above, they can directly eliminate invading pathogens by phagocytosis (32). This type of activation is also referred to as classical activation and results in the polarization of macrophages into the M1 phenotype with the aforementioned pro-inflammatory effector functions. On the other hand, macrophages are also actively involved in the resolution of the inflammatory response, repair processes and restoration of tissue homeostasis. Again, they can be induced to polarize into a secondary phenotype by stimuli such as IL-4 or phagocytosis of apoptotic neutrophils. In contrast to the pro-inflammatory M1 species, this results in anti-inflammatory, pro-regenerative M2 macrophages that release factors such as transforming growth factor-β (TGF-β), IL-10 and lipoxins. This is known as alternative activation (32, 39).

Thus, monocytes and macrophages are not only effector cells of the non-specific immune response, but also fulfil immunoregulatory functions and serve as a link to specific defense mechanisms during the inflammatory response (32).

#### 3.1.6 Lymphocytes

Lymphocytes can be subdivided morphologically into the smaller B and T cells and the larger natural killer (NK) cells (31). They have a rounded, dark nucleus with a very narrow cytoplasmic border (**Figure 3E**). Plasma cells, a differentiated form of B cells that produce antibodies, are unique in that they have an oval shape with an eccentrically positioned nucleus (**Figure 3F**). NK cells are larger than B and T cells and have a wider cytoplasmic rim containing large azurophilic granules (34).

B and T cells serve as the main carriers of the adaptive immune system. With the help of an antigen receptor, they can bind a specific foreign antigen and precisely initiate targeted immunological defense mechanisms. The B or T cell receptor, which is known to exist in numerous molecular forms, enables the recognition of virtually all potential antigens in the environment. This receptor specificity fundamentally distinguishes lymphocytes from the previously described immune cells (31). Although part of adaptive immunity, lymphocytes also participate in the innate inflammatory response and interact with respective immune cells.

T cells, along with macrophages, are important resident immune cells in the dental pulp. Even in healthy pulp tissue, they constitute a significant proportion, which increases during inflammation (18, 37, 38). T cells do not have the ability to recognize antigens directly, but their recognition depends on the presentation of antigen fragments by specialized cells such as macrophages and dendritic cells (40). It is worth noting that when activated, T cells produce cytokines that recruit and stimulate macrophages, leading to increased antigen presentation (4).

In general, there are several main types of T cells. Helper T cells (CD4+) orchestrate the activation of other cells involved in the immune response through a variety of interleukins. They also induce the differentiation of B cells into antibody-producing plasma cells (4). In terms of defense, cytotoxic T cells (CD8+) primarily target cells infected with a virus or other intracellular pathogens. They can eliminate these cells directly through their cell-mediated cytotoxicity and also activate macrophages (41). In the dental pulp, helper and cytotoxic T cells are present not only during the inflammatory response but also in healthy tissue (18, 38), although their numerical ratio has been reported inconsistently (37, 42). Furthermore, there are regulatory T cells (Treg), a specialized CD4+ T cell subset formerly known as suppressor T cells, that arise as a distinct lineage and inhibit the effector functions of other T cell types (43). Tregs have also been described in healthy pulp tissue and may play an important role in the modulation of pulpitis (18, 44).

Unlike T cells, B cells can recognize antigens directly through their B cell receptors. In their mature form as plasma cells, their primary function is to produce antibodies that contribute to the specific humoral immune response (41). Very low numbers of B cells and plasma cells are found in the pulp of healthy teeth (18, 38, 45). However, when the pulp encounters pathogens and leukocytes are recruited from the circulation, the number of B cells in the pulp tissue increases (19).

NK cells possess both antibody-dependent cytotoxicity, where they bind to antibody-coated target cells and induce their lysis, and NK activity, which is a direct mechanism leading to the destruction of virus-infected or transformed cells (46). Their cytotoxic potential is attributed to components stored in granules, including lysing enzymes and perforin (35). In both healthy and inflamed pulp tissue, NK cells represent a similarly small proportion of the leukocytes, with a suggested role in immune surveillance (18).

#### 3.1.7 Dendritic cells

Major histocompatibility complex (MHC) class II-positive dendritic cell (DC)-like cells, which also express macrophage lineage markers, have been found in the peripheral pulp area of human teeth with caries (47). DCs recognize microbes through pathogen-associated receptors in peripheral tissues, secrete various cytokines and chemokines to initiate innate immunity, and migrate to regional lymph nodes (16). There, pulp-derived DCs present captured microbial antigens to naive T cells, resulting in effector T cell recruitment to the pulp and initiation of the adaptive immune response (48).

### 3.2 Specific cells in the pulp tissue

Metabolically active bacteria in a carious lesion release products that induce inflammatory responses and morphological changes in the pulp even before the microbes themselves reach the pulp cavity. Bacterial metabolites and mediators pass through the tubular dentin towards the pulp, activating pro-inflammatory signaling pathways and promoting odontoblasts, pulp fibroblasts and tissue-resident immune cells to respond to microbial threats. However, due to their initial dissemination in the dental hard tissues, the bacteria cannot be directly targeted by immune cell phagocytosis until they infiltrate the pulp tissue (8).

#### 3.2.1 Odontoblasts

Due to their position at the pulp-dentin interface and their cell processes extending into the dentinal tubules, odontoblasts are the first cells to encounter invading microbes and their toxins. As a pseudo-epithelial layer, they form a partially impermeable barrier similar to other epithelia (48).

Activation of TLRs and NLRs induces various defense mechanisms in odontoblasts. Through their cell processes, they release antimicrobial peptides, such as β-defensins, into the dentinal tubules. In addition to their bactericidal effect, these peptides also have modulating functions within the immune response. They can stimulate the production of pro-inflammatory cytokines and chemokines in immune cells, as well as the maturation of dendritic cells and the differentiation of macrophages (8). Furthermore, e.g. upon exposure to lipoteichoic acid (LTA) or lipopolysaccharide (LPS), odontoblasts can release chemokines that recruit additional dendritic cells into the odontoblast layer (16). Finally, odontoblasts and dendritic cells work together to trigger the inflammatory response by releasing inflammatory mediators, such as cytokines and prostaglandins, and recruiting T cells, B cells, macrophages and neutrophil granulocytes via chemotactic signals (8). Odontoblasts also produce vascular endothelial growth factor (VEGF) after stimulation by gram-positive bacteria, leading to a significant increase in vascular permeability and angiogenesis (49). In addition, cariogenic bacteria induce the production of lipopolysaccharide binding protein (LBP), an acute phase protein, in odontoblasts. This protein neutralizes bacterial cell wall components, reducing pulp stress and limiting the inflammatory response (50).

#### 3.2.2 Fibroblasts and stem cells

Fibroblasts make up the majority of the cell population in the dental pulp. They are spindle-shaped cells with long, thin and often branched processes. Their nuclei typically have an elongated morphology and are rich in heterochromatin. The cytoskeleton of fibroblasts is highly specialized and they have the ability to move actively (49). Fibroblasts, key connective tissue cells, are responsible for synthesizing and organizing the extracellular matrix. During inflammation, they are inevitably affected by the ongoing processes. Through TLRs and NLRs, fibroblasts recognize pathogens and orchestrate the accumulation and differentiation of leukocytes through cytokine secretion. Additionally, pulp fibroblasts can synthesize all components of the complement system (50). The local production of complement proteins can amplify the inflammatory reaction, kill cariogenic bacteria through the formation of the membrane attack complex (MAC) C5b-9, and promote phagocytosis through opsonization. Moreover, fibroblasts have the capacity to facilitate nerve sprouting in the context of regenerative processes by activating the complement system and producing neurotrophic factors (51).

Another relevant cell population within the dental pulp is mesenchymal stem cells (MSCs), specifically dental pulp stem cells (DPSCs), which possess key stem-like properties such as self-renewal and multi-lineage differentiation potential (52). Like MSCs in most organs, DPSCs reside in the perivascular niche of the dental pulp in a quiescent state. Once activated, these undifferentiated cells can migrate to the site of injury, where they have the potential to replace damaged odontoblasts and synthesize new dentin (14). Self-renewal ensures that each DPSC division produces at least one new stem cell, allowing undifferentiated stem cells to remain. In addition, MSCs have immunomodulatory functions, such as promoting CD4+, CD8+ and Treg proliferation, suppressing cytotoxic T cell production, and inhibiting B cell differentiation. They can also alter the immunophenotype of macrophages to enhance tissue regeneration at the site of inflammation (53).

Both, fibroblasts and the morphologically similar DPSCs are actively involved in ending the inflammatory response by creating an anti-inflammatory tissue environment after a successful immune defense. This shift in secreted mediators stops the recruitment of leukocytes, facilitates their clearance and initiates repair processes. However, when this balance is disrupted, as in chronic inflammation, it can result in persistent leukocyte recruitment and progressive tissue fibrosis (31).

## 4 Inflammatory signaling in the pulp

### 4.1 Common immune mediators

Various signaling molecules play a central role in the inflammatory cascade in the dental pulp. They are known as inflammatory mediators and can be of either cellular or plasma origin. Whereas mediators of cellular origin are stored intracellularly in vesicles and released by exocytosis or synthesized in response to a stimulus, plasmatic mediators initially circulate in the blood as inactive precursors and are mainly synthesized in the liver (4, 54). Based on their biochemical properties, inflammatory mediators can be divided into several groups: vasoactive amines, vasoactive peptides, complement fragments, lipid mediators, platelet-activating factors, cytokines, chemokines, proteases and reactive nitrogen and oxygen species (**Table 2**) (26, 54).

**Table 2:** Cell- and plasma-derived mediators of inflammatory responses (107, 108).

Mediator	Function	Sources	
Cell-derived			
Histamine	Vasodilation, increase of vascular permeability	Mast cells, basophils, platelets	
Serotonin	Vasoconstriction	Platelets	
Prostaglandins	Vasodilation, pain, fever	Leukocytes, e.g. mast cells	
Leukotrienes	Increase of vascular permeability, leukocyte adhesion and activation	Leukocytes, e.g. mast cells	
Lipoxins	Inhibition of chemotaxis and diapedesis, inhibition of proinflammatory cytokine release, pain reduction	Leukocytes	
Resolvins	Inhibition of granulocyte transendothelial migration, promotion of regenerative processes, pain reduction	Leukocytes	
Maresins	Inhibition of neutrophil granulocyte recruitment, promotion of regenerative processes	Macrophages	
Platelet-activating factor	Vasodilation, increase of vascular permeability, leukocyte adhesion, chemotaxis, degranulation	Leukocytes, e.g. mast cells	
Cytokines	Local endothelial activation, fever, pain, anorexia, hypotension, decreased vascular resistance (shock)	Endothelial/epithelial cells, leukocytes, fibroblasts	
Chemokines	Chemotaxis, leukocyte activation	Endothelial/epithelial cells, leukocytes	
ROS/RNS	Toxicity against viral and microbial pathogens, tissue damage	Leukocytes	
Substance P	Vasodilation, leukocytes recruitment and activation	Peripheral nerve fibers	
Plasma-derived			
Kinins	Vasodilation, increase of vascular permeability, pain	Liver
Complement	Leukocyte chemotaxis and activation, vasodilation, opsonization	Liver
Proteases	Endothelial activation, leukocyte recruitment	Liver (endogenous) or microorganisms (exogenous)

#### 4.1.1 Vasoactive amines

The biogenic amines histamine and serotonin, the latter also known as 5-hydroxytryptamine (5-HT), have complex effects on the vasculature. In arterioles and venules, histamine acts on endothelial cells to cause vasodilation and increase vascular permeability. In larger vessels such as arteries and veins, it causes vasoconstriction via its effect on smooth muscle cells (54, 55). 5-HT also has a regulatory effect on the immune system. It is involved in the activation of T cells, the recruitment of neutrophils and the modulation of cytokine secretion by macrophages (56).

#### 4.1.2 Vasoactive peptides

In addition to their effect on nociception, neuropeptides stored in vesicles, such as substance P, released from peripheral nerve endings of sensory nerve fibers, cause vasodilation, increase vascular permeability and lead to activation and recruitment of leukocytes (57). There are also circulating precursors of vasoactive peptides that must be activated by proteolysis, such as the kinin-kallikrein system. Activation of kallikrein by Hageman factor leads to the formation of various kinins, including bradykinin. After tissue injury, bradykinin is one of the first inflammatory mediators to be activated and is important in initiating the acute inflammatory response. It is vasodilatory, increases endothelial permeability and stimulates leukocyte migration (26, 32).

#### 4.1.3 Complement fragments

The recognition of pathogens leads to the activation of complement proteins, a major initiator of the inflammatory response. In addition to liver-derived complement factors, which are essential for broad systemic immunity, locally produced complement proteins are essential for enhancing a rapid and targeted immune response directly at the site of infection or injury (58). The biologically active fragments C3a, C4a, and C5a of the complement proteins C3, C4, and C5 are known as anaphylatoxins. However, the status of C4a as an anaphylatoxin is controversial because its efficacy in the human organism has not been clearly established. These fragments have a chemotactic effect on granulocytes, macrophages and mast cells, activating them and leading to the release of other inflammatory mediators (32). The C3b fragment is known as an opsonin and has a pro-phagocytic effect on neutrophils and macrophages as part of the acute inflammatory response (4, 54).

#### 4.1.4 Lipid mediators

Lipid mediators are derivatives of polyunsaturated fatty acids that can have both pro- and anti-inflammatory effects. The eicosanoids, metabolites of C20 fatty acids such as arachidonic acid, are an important group (54). They include prostanoids (prostaglandins and thromboxanes), leukotrienes and lipoxins.

Prostaglandins cause vasodilation at the site of inflammation. In combination with leukotrienes and other mediators such as bradykinin and histamine, they also increase vascular permeability (32, 54). They are also important in nociception, leading to hyperalgesia and allodynia by sensitizing peripheral and central pain transmission and processing (59), and are involved in the development of fever (60). However, prostanoids can also initiate anti-inflammatory signaling pathways and have complex regulatory effects on cells of the innate and adaptive immune systems (61).

Leukotrienes also have effects on the vasculature in that the cysteinyl leukotrienes C4, D4 and E4 increase the permeability of post-capillary venules. In addition, leukotriene B4 has a potent chemotactic effect on neutrophil granulocytes (55).

Lipoxins, on the other hand, are anti-inflammatory and inflammation-dissolving lipid mediators. Lipoxin A4 and B4 act in the opposite way to leukotrienes and not only stop chemotaxis and diapedesis of neutrophil granulocytes but also inhibit the release of pro-inflammatory cytokines (31, 62). In addition, lipoxins reduce inflammatory pain and induce non-phagocytic phagocytosis of apoptotic neutrophil granulocytes (63, 64).

Besides the arachidonic acid-derived lipoxins, there are other more recently discovered families of inflammation-dissolving lipid mediators formed from long-chain omega-3 fatty acids: resolvins, protectins and maresins. Resolvins and protectins reduce the number of neutrophil granulocytes at the site of inflammation by blocking their transendothelial migration from blood vessels into tissue and stimulating their efferocytosis at the site. They also have an antagonistic effect on leukotriene B4 and promote tissue regeneration (65). Resolvins may also attenuate inflammatory pain and have an analgesic effect (66). Maresins (macrophage mediators in resolving inflammation) have a strong inhibitory effect on the recruitment of neutrophil granulocytes. They increase the efferocytosis of macrophages, inhibit the formation of leukotriene B4 (67, 68) and also promote tissue regeneration.

#### 4.1.5 Platelet-activating factors

PAFs are also lipid mediators and have broad pro-inflammatory effects. On the one hand, they lead to platelet activation and aggregation and promote the diapedesis of neutrophil granulocytes in the extracellular space. In addition, PAFs increase vascular permeability and are involved in inflammatory hyperalgesia. Their biosynthesis leads to the release of arachidonic acid and ultimately to an increased production of eicosanoids (31, 55).

#### 4.1.6 Cytokines and chemokines

Cytokines, small proteins released by cells, regulate the proliferation, differentiation and function of the various cells of the innate and adaptive immune systems following exposure to a pathogen. They ensure that these cells communicate with each other and coordinate their actions in order to mount a targeted immune response. At the same time, they regulate this defense response to prevent disproportionate damage to the organism and, once the cause has been successfully eliminated, to initiate regenerative processes and ultimately restore tissue homeostasis. This makes them key mediators of both acute and chronic inflammatory responses (32).

More than 300 different cytokines have been described (69) and they can be divided into three groups according to their targets: growth factors, hematopoietins and cytokines. They act on the cells that release them (autocrine), on neighboring cells (paracrine) and on more distant cells via the bloodstream (endocrine). They elicit pleiotropic activities, meaning that they are able to induce a wide spectrum of functional responses in a variety of cell subsets. Their interaction may be synergistic or antagonistic (31). Important cytokine families of the immune system include interleukins (IL), interferons (IFN) and the TNF superfamily.

Important proinflammatory cytokines include IL-1β, TNF-α and IL-6 (69). IL-1β has a wide range of pro-inflammatory effects and acts as endogenous pyrogen. It can induce the production of prostaglandin E2 (PGE2) and PAF and leads to vasodilation with increased vascular permeability. In addition, IL-1β activates endothelial cells to recruit leukocytes from the bloodstream into the tissue (32, 70).

TNF-α leads to the activation of several pro-inflammatory signaling pathways, including the activation of NF-κB. By stimulating the production of vasoactive mediators such as prostanoids, TNF-α has a vasodilatory effect and also increases vascular permeability via direct and indirect bradykinin-mediated effects. In addition, TNF-α facilitates vascular permeability by inducing the expression of specific adhesion molecules in endothelial cells, which facilitates the recruitment of leukocytes to the site of infection (71).

IL-6 leads to the release of acute phase proteins such as C-reactive protein (CRP) in the liver during the initial phase of the inflammatory response (72). It regulates the extent of inflammation by inhibiting the production of further pro-inflammatory cytokines and limiting the acute infiltration of neutrophil granulocytes into tissues (73). At the same time, IL-6 promotes the recruitment of macrophages and regulates the differentiation of B and T cells, playing a crucial role in the transition from a non-specific to a specific immune response, thus providing a link between the innate and acquired immune systems (74).

The most important anti-inflammatory cytokines are IL-10 and TGF-β (54). IL-10 is the most potent immunosuppressive cytokine. It inhibits the release of pro-inflammatory cytokines and the function of activated macrophages, controlling non-specific defense in a negative feedback loop. It also suppresses the ability to present antigens, the expression of MHC class II proteins and thus antigen-induced T cell activation (75).

TGF-β is classically regarded as both a pro-inflammatory and a regenerative cytokine, inducing cessation of the acute inflammatory response and initiation of repair processes, respectively. While it inhibits T and B cell proliferation and differentiation and reduces TNF-α production (76), TGF-β also has pro-inflammatory effects, for example by differentiating T cells into Th17 cells (77).

Chemokines are a specific family of structurally related and chemotactically active cytokines. They control the migration of leukocytes to the site of inflammation and can be produced by various tissue cells, such as epithelial and endothelial cells. Leukocytes themselves can also produce chemokines in a positive feedback loop (32). However, they also have other functions, such as T helper cell differentiation, hematopoiesis and angiogenesis (31, 69). The chemokine CXCL8 (or IL-8), with its pronounced chemotactic and activating effect on neutrophils, is of major importance in the acute inflammatory response (78).

#### 4.1.7 Proteases

Proteases are not only involved in processes of debris removal and tissue remodeling at the site of inflammation, they also have extensive functions in the inflammatory response and should be considered as signaling molecules. They can originate from invading microbes (exogenous) or be endogenous. Through receptors, including protease-activated receptors (PAR), proteases trigger extensive pro-inflammatory effects such as leukocyte activation, stimulation of the release of cytokines and other mediators, increase in microvascular permeability and hyperalgesia. Through proteolysis, they can also directly activate or inactivate cytokines and chemokines such as IL-1, IL-6, TNF-α, IL-8, facilitate the transendothelial passage of leukocytes and have a general antimicrobial effect (32).

#### 4.1.8 Reactive nitrogen and oxygen species

RNS and ROS are small nitrogen and oxygen compounds that are characterized by a special chemical reactivity as radicals. In the context of inflammatory responses, their direct toxic effect against viral and microbial pathogens is often the focus. Furthermore, they regulate and modulate the overall immune response, exerting context-dependent pro- or anti-inflammatory effects. They also interact with the NF-κB signaling pathway in a variety of ways (79).

Nitric oxide (NO), a well-known RNS, has a regulatory control function over lymphocytes, especially T cells, in addition to its potent vasodilatory and antimicrobial effects (80). The production of NO is strongly induced by proinflammatory cytokines and may contribute to the resolution of inflammation in a negative feedback loop. This occurs, for example, through inhibitory interactions with NF-κB signaling pathways and by inhibiting T cell proliferation. It also suppresses leukocyte recruitment by inactivating chemokines and inhibiting their generation, and by limiting neutrophil adhesion to the endothelium and transendothelial migration. In addition, apoptosis of already recruited neutrophil granulocytes is stimulated (80).

### 4.2 Signaling cascade in the context of pulpitis

Increased levels of the classic inflammatory cytokines IL-1β, IL-4, IL-6, IL-8, IL-10 and TNF-α can be detected in inflamed pulps in comparison to healthy pulps (85). The signaling molecules expressed by odontoblasts also play a crucial role. For example, increased formation of NO and increased expression of adhesion molecules for leukocyte recruitment, such as selectins, are observed particularly in the odontoblast layer (14, 86). In addition, odontoblasts produce chemokines (CCL2, CXCL1, CXCL2, CXCL8/IL-8, CXCL10) immediately after recognizing a pathogen to recruit effector cells of the inflammatory response (87).

### 4.3 Dentin matrix proteins as mediators

During tooth development, differentiated odontoblasts produce a matrix of collagen that is subsequently mineralized to form dentin. They also synthesize a large number of signaling molecules that remain embedded in dentin, bound to proteoglycans, glycoproteins or collagen. These bioactive molecules are known as dentin matrix proteins and are fundamental to the pulpal inflammatory response. They are released by caries-induced demineralization of dentin and can act as pro- or anti-inflammatory mediators. Even at low concentrations, they can be chemotactic, promote angiogenesis, stimulate proliferation and differentiation of progenitor cells and influence mineralization processes (8, 88). Specifically, this group includes matrix mineralization-associated proteins, growth factors, cytokines, neuropeptides, neurotrophic factors and components of the complement system (88–91). TGF-β1 is the most abundant growth factor in dentin, but bone morphogenetic protein (BMP)-2, platelet-derived growth factor (PDGF), placental growth factor (PIGF), epidermal growth factor (EGF) and angiogenesis factors such as basic fibroblast growth factor (bFGF) and VEGF are also bound within the dentin matrix (88, 92).

### 4.4 Microenvironmental factors

The inflammatory response in the dental pulp to caries may also be influenced by a number of microenvironmental factors, including the microbial flora, tissue dynamics, salivary composition and systemic health conditions.

As caries progresses, the composition of the biofilm evolves significantly, particularly in deep carious lesions. As oxygen availability and nutrient supply vary with cavity depth, the microbiota shifts from an aerobic and saccharolytic flora to an anaerobic and proteolytic flora. These associated bacteria release specific metabolites that penetrate the dentinal tubules and trigger immune responses. Thus, microbial changes in caries may influence the recruitment and activation of immune cells such as neutrophils and macrophages that respond to bacterial by-products (23).

Inflammatory processes within the pulp tissue during caries infection lead to metabolic and vascular changes and create a hypoxic environment. Low oxygen levels activate hypoxia-inducible factors (HIFs), which promote the production of inflammatory cytokines such as TNF-α and IL-1β. Under hypoxia, macrophages are more likely to polarize towards the pro-inflammatory M1 phenotype, escalating inflammation and tissue damage (39). Neutrophils, in turn, produce ROS to fight infection, however, excessive production can cause oxidative damage to pulp tissue.

Saliva, which also may enter through carious defects, contains antimicrobial peptides (AMPs) such as defensins and histatins that can modulate the behavior of immune cells within the pulp. Furthermore, secretory immunoglobulin A (sIgA) in saliva plays a protective role by neutralizing pathogens before they reach the pulp (81).

In addition to local factors, systemic conditions can influence the oral microenvironment and immune response. For example, in people with diabetes, chronic hyperglycemia impairs immune cell function, particularly the activity of macrophages and neutrophils (8). This can either weaken the immune response to infection or cause excessive tissue damage in the pulp. Likewise, patients with autoimmune diseases may have increased inflammation in the oral cavity (82). Systemic inflammation in these individuals also appears to have the potential to alter immune signaling in the pulp (83).

## 5 Physiological and structural changes in pulpitis

### 5.1 Stages of inflammation

The inflammatory response can be divided into two basic types: acute and chronic inflammation. These two forms do not represent distinct phases with a strict sequence, but rather describe different modes of response to an inflammatory stimulus (2). While acute inflammation constitutes the initial response to injury or infection and involves rapid and non-specific mechanisms, chronic inflammation is a prolonged and often more specific response involving both innate and adaptive immune components. Both types of inflammation are tightly regulated processes that are critical for maintaining tissue homeostasis and defending against pathogens. In addition to differences in timing and specificity, acute and chronic inflammation also have distinct morphological and histological characteristics, which will be discussed in more detail below.

#### 5.1.1 Acute inflammatory reaction

**Figure 4:**
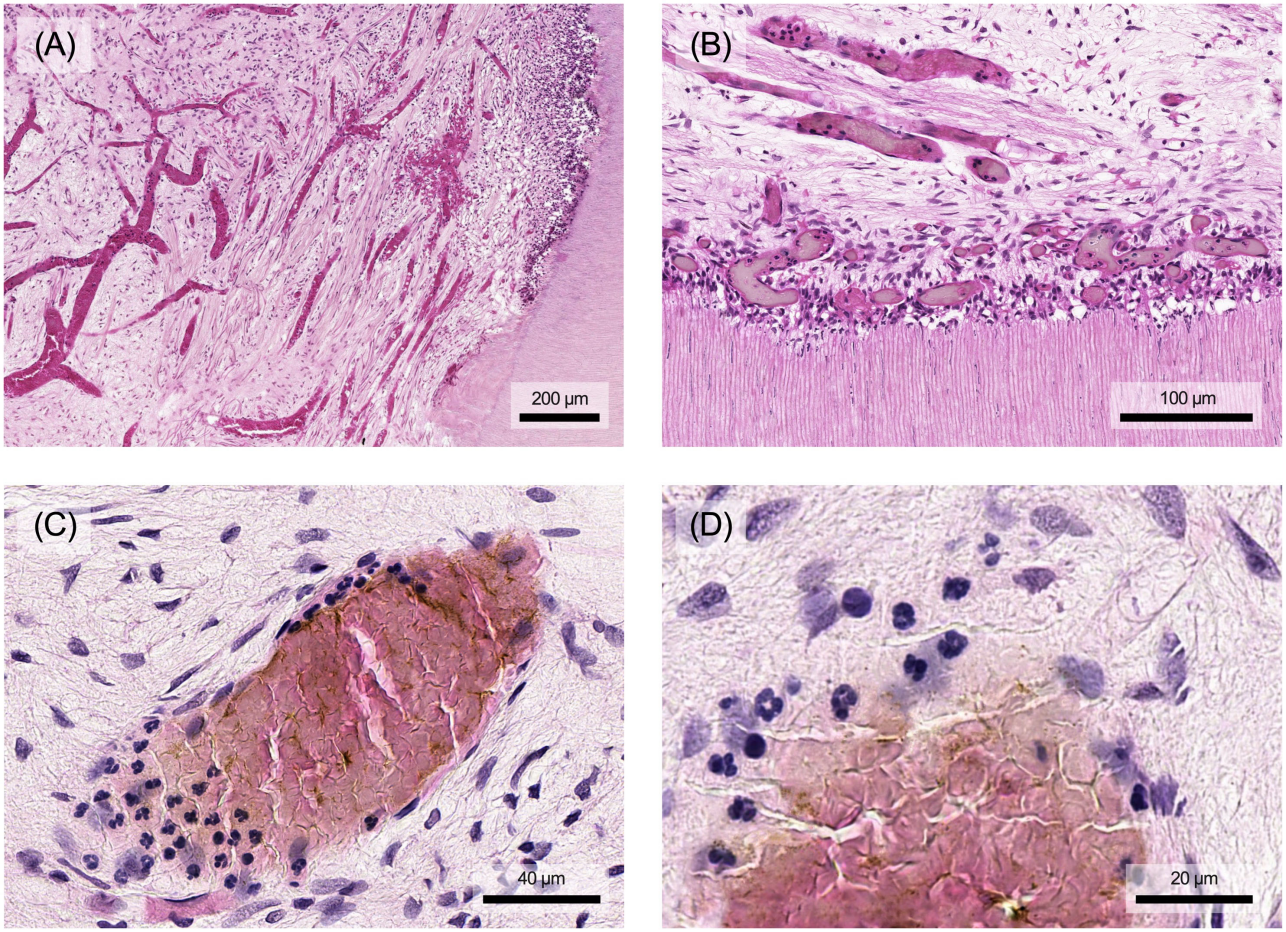
**(A)** In the course of acute pulpitis, vascularization of the tissue occurs with dilation of the vessels. **(B)** In particular, the subodontoblastic capillary plexus also shows significant vasodilation compared to healthy pulp tissue. The aim is to transport immune cells to the site of damage, e.g. neutrophils in the acute phase. **(C)** These first bind to the vessel wall and **(D)** then migrate through it into the tissue. Histological specimen from H. R. Stanley (13); staining: hematoxylin and eosin.

The morphological nature of acute inflammation involves two fundamental processes: vascular changes and cellular events (**Figure 4**). Vascular changes include dilation of blood vessels to increase blood flow and an increase in vascular permeability, which favors the movement of immune mediators. Cellular events consist of the migration of leukocytes from vessels, their accumulation in tissues and subsequent neutralization of pathogens (4).

Macroscopically, different patterns of acute inflammation emerge based on the composition of the resulting inflammatory exudate. Serous inflammation is characterized by the accumulation of cell-poor fluid in tissues, whereas fibrinous inflammation involves the leakage of larger molecules such as fibrinogen from vessels, forming typical fibrin deposits. Suppurative inflammation manifests as an exudate rich in neutrophils, resulting in the classic appearance of yellowish pus (4).

Acute inflammation can lead to three possible outcomes: complete histological and functional recovery when tissue damage is minimal and the trigger is successfully eliminated; healing by scarring or fibrosis with loss of function in tissues (repair); or transition to chronic inflammation when the inflammatory response cannot be adequately resolved due to persistent triggers or failure of the termination mechanism (4).

Vasodilation is one of the first stages of the acute inflammatory response (**Figures 4A, B**). Triggered by vasoactive agents such as histamine, prostaglandins and NO, it initially targets the arterioles, causing the capillary beds in the affected area to fill. This increased blood flow leads to the characteristic signs of redness and warmth. Shortly after vasodilation, there’s a rapid increase in microvascular permeability within venules and capillaries. This is primarily due to contraction of the endothelial cells, which increases the space between them, allowing greater passage of fluids and proteins. Factors such as histamine, bradykinin, leukotriene, substance P and PAF contribute to this process (4, 84). Along with the elevated hydrostatic pressure due to increased blood flow, this results in leakage of plasma and proteins into the surrounding tissues, leading to inflammatory swelling (54). In addition, these vascular changes affect not only the blood vessels but also the lymphatic system, promoting dilation to improve lymphatic drainage. This helps to remove inflammatory cells and debris, thus contributing to the resolution of inflammation (4).

The vascular changes described above are followed, with a slight delay, by an influx of leukocytes into the affected tissue. A hallmark of the acute inflammatory response is the substantial infiltration of neutrophilic granulocytes (**Figures 4C, D**). Vasodilation slows blood flow and alters hemodynamic forces, leading to the marginalization of leukocytes towards the vascular endothelium (4). Inflammatory mediators such as TNF-α, IL-1 and histamine activate endothelial cells and induce the expression of adhesion molecules on their surface (P- and E-selectins). These molecules then bind to specific surface molecules on neutrophile granulocytes, initiating their adhesion and rolling along the vessel wall (**Figure 4C**). This selectin-mediated rolling leads to further activation of leukocytes by proinflammatory chemokines presented on the endothelial cell surface (4, 31). Subsequently, integrins on the surface of leukocytes bind to inflammatory ligands on endothelial cells (e.g. intercellular adhesion molecule-1 [ICAM-1]), halting their rolling motion and firmly attaching them to the endothelium (4). Subsequent diapedesis, the process of crossing the vessel wall, occurs by two different mechanisms (**Figure 4D**). Leukocytes can migrate between endothelial cells by paracellular routes facilitated by specific molecules such as platelet-endothelial cell adhesion molecule-1 (PECAM-1). Alternatively, electron microscopic observations suggest that leukocytes can also transmigrate through endothelial cells (31, 85). They then penetrate the adjacent basement membrane by secreting collagenases, allowing them to enter the tissue. Once in the tissue, leukocytes follow gradients of chemotactic mediators such as IL-8 and leukotriene B4 to their destination (4).

#### 5.1.2 Chronic inflammatory reaction

**Figure 5:**
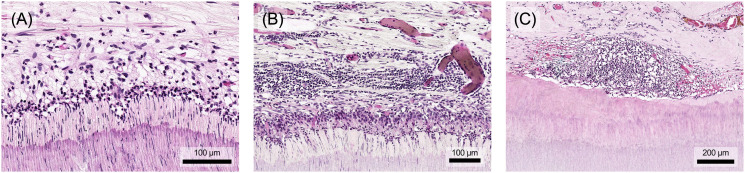
The inflammatory response of the dental pulp changes structurally depending on the extent and persistence of the stimulus. **(A)** Initially, there is a local infiltration of single immune cells. **(B)** The immune cell infiltrate spreads in multiple layers throughout the tissue and **(C)** may form an extensive but localized focus in the pulp, which is ultimately accompanied by local destruction of tissue components. Histological specimen from H. R. Stanley (13); staining: hematoxylin and eosin.

Chronic inflammation can result from an acute reaction in response to a persistent pathogen. However, it can also develop *de novo* in the absence of preceding acute responses, for example in viral infections. Prolonged exposure to exogenous and endogenous irritants can also induce chronic inflammation, such as silica in pulmonary silicosis or plasma lipids in atherosclerosis (4). Chronic inflammation also occurs in tissues affected by autoimmune diseases (4, 32). In contrast to acute inflammation, chronic inflammation is histologically characterized by the infiltration of tissue with macrophages, lymphocytes and plasma cells. As the stimulus continues, the initial local accumulation of immune cells in the pulp becomes increasingly widespread (**Figure 5**). Additionally, significant tissue damage can be observed alongside ongoing repair processes. These repair processes are characterized by angiogenesis and fibrosis (4).

Macrophages dominate the cellular composition at sites of chronic inflammation, playing a dual role in initiating repair processes and contributing significantly to tissue damage. Recruitment of B and T cells to sites of inflammation is facilitated by shared adhesion molecules and chemokines. Reciprocal interaction between T cells and macrophages establishes an inflammatory focus where continuous stimulation of both cell types perpetuates the chronic inflammatory response (4, 32).

There are generally two patterns of cellular infiltrate distribution in chronically inflamed tissue. Usually, the infiltrate is relatively evenly dispersed throughout the tissue. However, in certain cases of chronic inflammation there is a specific morphological manifestation known as granulomatous inflammation (54). This response is triggered by persisting pathogens or inert particles and results in nodular clusters of activated macrophages and T cells surrounding the causing agent. Granulomas can be categorized into immunological and foreign body types based on the nature of the triggers. Foreign body granulomas form around non-antigenic irritants that cannot be phagocytosed and initiate the defense response via the Hageman factor. In contrast, immunological granulomas develop in response to pathogens capable of inducing a T cell-mediated immune response, with cellular components surrounding the pathogen in a concentric fashion (54, 86).

#### 5.1.3 Neurogenic inflammation

Activation of the neural system is an integral part of the inflammatory response. Within this process, the peripheral nervous system plays a direct and proactive role in the regulation of both innate and adaptive immune mechanisms and is thus a vital component of the body’s defense mechanisms (87). Afferent nerve fibers within peripheral sensory nerves transmit action potentials to central relay stations in the spinal cord or brainstem. Some of these afferent fibers not only transmit these signals orthodromically but can also induce an inflammatory response throughout the innervation area of the nerve endings in the opposite direction - this is known as neurogenic inflammation. As almost all tissues in the human body are innervated by nociceptive afferents, neurogenic inflammation can occur ubiquitously (88). Predominantly unmyelinated sensory nerve fibers can be activated by various noxious stimuli, including physical (thermal, mechanical, electrical) and chemical factors.

This activation leads to the release of neuropeptides, in particular substance P (SP) and calcitonin gene-related peptide (CGRP), from nerve endings. SP directly induces vasodilation and increases the permeability of surrounding blood vessels, resulting in plasma extravasation. In addition, SP induces vascular effects indirectly by stimulating mast cells and triggering the release of mast cell mediators such as histamine. The released CGRP also triggers intense vasodilation (32, 88). Besides their vascular effects, mediators released by sensory neurons also act as chemoattractant for neutrophils, macrophages and T cells, influencing their activation and differentiation (87). There is also evidence that neuropeptides have direct antimicrobial properties and thus contribute directly to the body’s defense against microorganisms (89). Proinflammatory mediators such as TNF-α and IL-1β can sensitize nociceptive nerve endings within the inflammatory milieu, enhancing pain perception and the neurogenic inflammatory response. In addition, nociceptive neurons also express PRRs, such as TLRs. Stimulation of these receptors also sensitizes the neurons, suggesting that some of the pain experienced during infection results from direct activation. Furthermore, it indicates that these neurons may initiate inflammatory defense mechanisms as an immediate response to microbial invaders (87, 88).

### 5.2 Characteristics of the pulpal inflammatory response

The inflammatory response is a vital defense mechanism in vascularized tissues such as the dental pulp. Although it follows universal principles applicable to all tissues, pulpitis is uniquely influenced by local factors arising from the anatomy, physiology and microenvironment of the oral cavity (15, 99). The fact that the tissue is completely surrounded by hard tissue and therefore spatially confined is certainly a unique feature. Due to tubular permeability and cellular nociception in the dentin, the pulp tissue reacts early to pathogenic external stimuli, such as bacteria from caries, which have not yet come into direct contact with the tissue. Characteristic structural changes to external stimuli are neurovascular adaptations in the course of the acute or chronic inflammatory response and the formation of dentin as a defense mechanism.

#### 5.2.1 Neuro-vascular changes

An early indicator of the immune response within the pulp is a change in its microcirculation (14). The pulp is a highly innervated tissue, and the mechanisms of neurogenic inflammation are of significant importance in modulating its inflammatory response (99). Upon stimulation, sensory nerves release several neuropeptides, including SP, CGRP, vasoactive intestinal polypeptide (VIP), neuropeptide Y (NPY), and calcitonin (CT), leading to recruitment of inflammatory cells, vasodilation and increased permeability of the microvascular system (8). The result of increased tissue perfusion is edematous swelling in the confined pulp chamber, which stresses the venous and lymphatic vessels and can lead to pulp damage and even necrosis. In addition, increased tissue pressure promotes the outward flow of dentin fluid, protecting the pulp from pathogen invasion (18, 100). Furthermore, inflammatory processes and sensory nerve injury can stimulate the growth of new nerve endings into the remaining healthy pulp tissue, thereby amplifying the inflammatory response through the additional release of neuropeptides from these newly formed branches (8).

#### 5.2.2 Tertiary dentin formation

**Figure 6:**
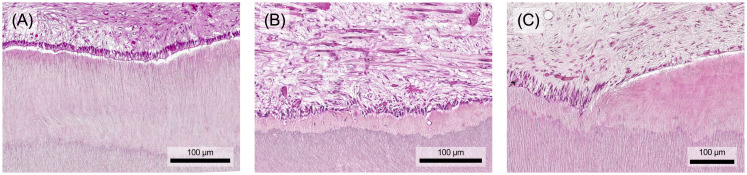
**(A)** In response to external stimuli, the odontoblasts form reactionary dentin, which is characterized by its still tubular structure. **(B)** If the odontoblast layer is destroyed mechanically or by excessive stimuli, replacement cells form so-called reparative dentin, which contains hardly any tubuli and is characterized by cell inclusions. **(C)** Both types of tertiary dentin may be mixed or found in close proximity. Histological specimen from H. R. Stanley (13); staining: hematoxylin and eosin.

A key aspect of pulpal defense and wound healing involves the sclerosis of dentin tubules and the formation of tertiary dentin, creating a mineralized barrier between the pulp and areas of bacterial invasion or injury. Tertiary dentin manifests as either reactionary or reparative dentin (**Figure 6**). These forms differ both in the cells responsible for their production and their morphological structure. Reactionary dentin is formed in response to mild stimuli that cause the odontoblasts to produce tubular dentin (**Figure 6A**). In contrast, reparative dentin forms when severe stimuli destroy odontoblasts. Mineralization is then facilitated by secondary odontoblasts, arising from pulp stem cell niches, which differentiate into mineralizing cells. Reparative dentin typically exhibits an atubular, irregular (osseous) structure, often with cellular inclusions (**Figure 6B**). The two types of tertiary dentin, reactionary dentin and reparative dentin, appear depending on the stimulus and can also occur as mixed forms and close to each other (**Figure 6C**) (8).

As described above, the pulp-dentin-complex has significant regenerative potential in response to injury or infection. While exacerbated inflammatory processes result in tissue degradation and molecular signaling that inhibits regeneration, low-grade inflammation may promote regenerative mechanisms in tertiary dentin secretion. Secretory activity is finely modulated by locally derived growth factors, neuropeptides, cytokines and chemokines released from the dentin matrix and by resident pulp cells, immune cells and neurons within the pulp tissue (90). A balance in favor of tissue regeneration is required, which can only occur when infection and inflammation are under control. Otherwise, pulp inflammation overrides the regenerative processes and the pulp tissue lacks the cellular capacity to secrete tertiary dentin in a controlled manner. Interestingly, studies also show that inflammatory signaling molecules, e.g. TNF-α and ROS, at low concentrations as they occur in early caries, upregulate the dentin secretory activity of odontoblasts and induce the differentiation of progenitor cells, e.g. through the p38 MAPK pathway (90, 91).

## 6 Termination of inflammatory processes in the pulp-dentin-complex

### 6.1 General steps towards recovery

The inflammatory response is a vital defense mechanism for the body. However, its effectiveness must be balanced with the need to limit damage to the body’s tissues. Regulation is essential to prevent excessive self-damage. This control occurs at several levels, involving triggers, detectors, mediators and the specific sites where these mediators act (92). An inflammatory response that has been successful in terms of defense ideally ends with the complete cessation of inflammatory processes and the restoration of tissue homeostasis. Essential to this is the neutralization and removal of the initial pathogen. This involves the recruitment and aggregation of neutrophil granulocytes by a combination of pro-inflammatory mediators. A key aspect of the resolution of inflammation is the clearance of accumulated leukocytes, a process that is tightly regulated at multiple levels, with lipid mediators playing an important role. During the acute phase of inflammation, the production of pro-inflammatory eicosanoids such as PGE2 and leukotriene B4 drives the recruitment of neutrophil granulocytes. However, the production of PGE2 also triggers a shift in metabolism towards anti-inflammatory lipoxins and the production of resolvins and protectins (93). This switch in the class of lipid mediators during the course of the inflammatory response demonstrates that leukocytes can define the termination of inflammation (94). By switching to lipoxins, resolvins and protectins, the neutrophil granulocyte recruitment is diminished and their apoptosis in the inflamed area is induced. In addition, macrophage migration increases along with their phagocytic activity, particularly in the clearance of apoptotic neutrophils. This process, known as efferocytosis, further stimulates macrophages to release cytokines such as IL-10 and TGF-β, thereby dampening the inflammatory response.

### 6.2 Termination of pulpitis

#### 6.2.1 Healing processes of the pulp

While the inflammatory response is essential for pulp defense and a prerequisite for pulp healing, its regulation and eventual resolution are critical for enabling tissue regeneration. The pulp is highly sensitive to pro-inflammatory signals, with low levels playing a key role in promoting regenerative processes such as cell recruitment, differentiation and tertiary dentin secretion. However, increased inflammatory signaling triggers a more intense immune response, which is necessary to clear infection (90, 91). Until the carious infection is controlled and pulpitis resolves, inflammation may prevent the restoration of tissue homeostasis, architecture and controlled tertiary dentin secretion. This can occur either through direct inhibition of regenerative signals or indirectly through tissue destruction. Specifically, in the context of tertiary dentinogenesis, the level of infection and inflammation is likely to determine whether dentin is secreted and whether it’s reactionary or reparative. It may be futile to initiate reparative dentin secretion if significant infection and inflammation persist, as these processes cause ongoing tissue damage (90).

**Figure 7:**
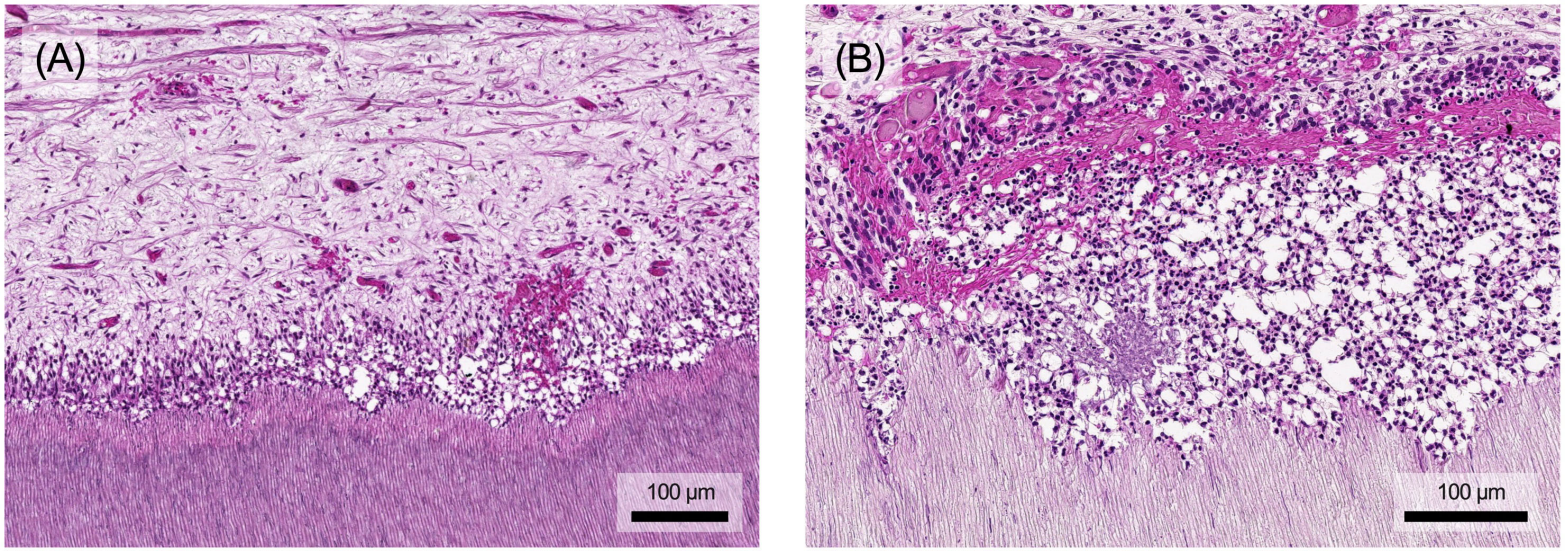
**(A)** The accumulation of inflammatory cells also causes structural damage to the pulp. The odontoblast layer is increasingly degraded and local bleeding may occur. **(B)** In addition to the destruction of the soft tissue in the pulp, prolonged stimulation may also lead to atypical vascular formations and dentin degradation, which in extreme cases can result in internal resorption processes. Histological specimen from H. R. Stanley (13); staining: hematoxylin and eosin.

The pulp's immune response employs various mechanisms to protect connective tissue from significant, irreversible damage (**Figure 7**). Proteases, for example, facilitate immune cell migration by degrading the extracellular matrix. However, prolonged or intense stimulation can lead to chronification, premature aging, decreased resistance or tissue degradation, opening the door for bacterial invasion of the pulp chamber and root canal system (8). Eventually, chronic pulp inflammation can result in internal inflammatory root resorption, where clastic cells erode dentin. Early detection of resorptive defects and prompt removal of the inflamed, often infected pulp tissue, followed by endodontic treatment, usually results in a good long-term prognosis (95, 96).

**Figure 8:**
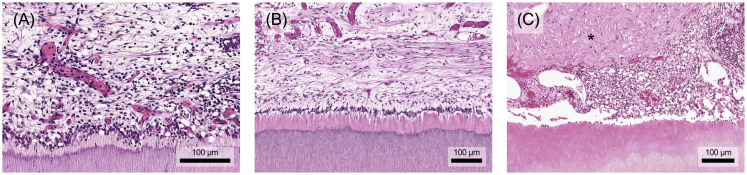
**(A)** The aim of healing after removal of the stimulus is for the immune cells to leave the tissue and for the original structure to be restored. **(B)** In the best case, the pulp-dentin-complex regenerates and a functional odontoblast layer remains. **(C)** However, a repair tissue with structural and functional limitations (marked with an asterisk) may also form during the healing process and fill the defect. Histological specimen from H. R. Stanley (13); staining: hematoxylin and eosin.

Removing the causative agent, e.g. by caries excavation, can resolve inflammation, neutralize toxins, activate anti-inflammatory pathways, and facilitate tertiary dentin formation (8). The depth of the carious lesion and the thickness of the remaining dentin protecting the pulp are crucial in determining the extent of inflammation. Lesions with less than 0.5 mm of remaining dentin usually trigger an extensive immune response (**Figure 8A**) (8, 97). These inflammatory processes are closely tied to tissue repair mechanisms, with mediators such as TGF-β, TNF-α, and bacterial components exerting concentration-dependent effects. While they promote repair at low concentrations (**Figure 8B**), their destructive impact increases at higher concentrations (**Figure 8C**) (8, 20). Depending on the stimulus intensity and tissue damage repair and healing might be unattainable, potentially leading to chronic inflammation and pulp necrosis (8).

#### 6.2.2 Stem cells in processes of repair and regeneration

Local mesenchymal stem cells, DPSCs, in their respective niches are critical for regular cell turnover and pulp repair and regeneration. They are recruited along chemotactic gradients to sites of tissue damage where they differentiate into mineralizing cells that form tertiary dentin. DPSCs are activated by inflammatory mediators, TLR signalling or immune cells such as macrophages (98, 99).

In addition to their progenitor potential, mesenchymal stem cells have extensive immunoregulatory functions, influencing both innate and adaptive immune processes. Depending on their microenvironment, they can play both pro- and anti-inflammatory roles within the immune cascade. In an inflammatory milieu with high levels of pro-inflammatory mediators (e.g. TNF-α) or TLR stimulation, stem cells adopt an immunosuppressive, anti-inflammatory phenotype and produce elevated levels of mediators such as PGE2, NO and TGF-β (100). In contrast, in an environment with low levels of inflammatory mediators, stem cells may differentiate towards a pro-inflammatory phenotype (101). In this role, they support early inflammation by recruiting neutrophils and enhancing their activity through cytokine release.

Once pro-inflammatory mediators reach a certain concentration, stem cells switch to an anti-inflammatory phenotype (101). In this capacity, their secreted mediators modulate lymphocyte differentiation, redirecting the immune response away from inflammatory effector T cells and toward regulatory T cells. They suppress dendritic cell maturation, reduce B cell activation and proliferation, and inhibit NK cell activity. In addition, they significantly stimulate macrophage polarization toward the anti-inflammatory and pro-regenerative M2 phenotype (101, 102). Thus, dental pulp stem cells actively contribute to the resolution of excessive inflammatory responses and initiate the transition toward restoration of tissue homeostasis (101, 103).

## 7 Future perspective

Microbial invasion resulting from dental caries or traumatic injuries affects a significant portion of the global population and can result in pulpitis and pain if untreated (9, 10, 109). Microbes and their toxins diffuse through dentinal tubules, triggering a localized host response in the pulp. Without proper treatment, the inflammation spreads, potentially leading to irreversible pulpitis, pulp necrosis and apical periodontitis. The disease progression involves a complex molecular interplay of various cell types within the pulp. Immune cells participate in both innate and adaptive immune processes, while tissue-specific cells such as odontoblasts, pulp fibroblasts, and DPSCs play multifaceted roles. Beyond their involvement in immunity through pathogen recognition and immunomodulation, odontoblasts or differentiated stem cells produce tertiary dentin to limit bacterial invasion and protect the pulp (12, 105).

Pulp healing requires elimination of infection and resolution of inflammation. While inflammation is essential for healing and tissue homeostasis, it can also cause collateral tissue damage. Inflammatory mediators such as cytokines, ROS and bacterial constituents can stimulate repair mechanisms in moderate concentrations but can also lead to chronic inflammation or acute exacerbation, resulting in necrosis. Thus, there is a delicate balance between pathogenesis and healing of the affected pulp that is controlled by a complex interplay of various cellular and acellular factors (19, 91).

Pulp has a high innate regenerative potential and resists carious infection by attempting to localize inflammation and prevent its spread. As structural changes appear to be spatially limited, traditional therapeutic assumptions have been increasingly challenged in recent years and minimally invasive, biologically based dental procedures are being developed (12, 19). The main goal is to preserve the vitality of the pulp tissue, at least in part, which traditionally would have been removed due to a lack of biological understanding, therapeutic options or adequate diagnostics. This can preserve tooth structure, allowing the tooth to remain functional for longer, and less invasive and targeted treatments can save time and money and reduce pain and discomfort for the patient (110).

However, there is still considerable uncertainty about the immunological processes and structural changes in the dental pulp at both the tissue and cellular levels. While the immune cellular composition in healthy and diseased states has been ambitiously outlined over the past decades, details of the specific functions of immune cells and their interactions with tissue-specific cells in the dental pulp remain unclear. Furthermore, the complexity of the inflammatory response and in particular its spatial and temporal progression is not yet understood. Especially recent immunological findings and methodological developments offer great opportunities to unravel this puzzle and shed light on previously unclear areas. A thorough knowledge of these processes and a defined correlation between structural changes and clinical symptoms are crucial for therapeutic decisions.

Clinical symptoms are the primary diagnostic tool for predicting pulpal disease and guiding treatment decisions. Traditionally, the pulp has been evaluated as a whole: preserved in cases of ‘reversible pulpitis’ and completely removed in cases of ‘irreversible pulpitis’, which is characterized by lingering thermal and spontaneous pain. It is well accepted today that structural changes are localized and do not affect the entire pulp to a certain degree, and that cases traditionally classified as irreversible may still be salvageable with respective treatment (11). Therefore, adaption of the diagnostic criteria for pulpitis and advances in diagnostics, such as specific molecular markers, play an important role in this context (11, 111).

From a therapeutic perspective, caries removal will remain the cornerstone of any treatment. In recent years, promising strategies have been described that use minimally invasive procedures to selectively remove caries, perform multi-session treatments or targeted amputations of irreversibly damaged pulp segments (110). These approaches offer clinical benefits and improved tooth survival. Currently, bioactive materials are applied after caries excavation to stimulate the pulp to form hard tissue, albeit in a rather unspecific manner (110). Future treatment strategies may become much more specific, pharmacologically intervening in complex defense processes to broaden the range of vital pulp therapies (19). Immunotherapeutic approaches to maintain pulp vitality could aim to specifically regulate or stop inflammatory processes, alleviate clinical symptoms and promote healing rather than cause irreversible tissue damage.

Advancing innovative approaches to clinical diagnostics and dental pulp treatment requires a thorough understanding of key clinical issues and the fundamental biology of the pulp-dentin-complex, including its immunological components in health, disease and healing. Close partnerships between practitioners and researchers are essential to move the field forward, generate new ideas and improve patient outcomes.
